# The structural and organizational aspects of human papillomavirus vaccine affecting immunization coverage in Europe: a systematic review

**DOI:** 10.1186/s12889-025-22343-w

**Published:** 2025-04-03

**Authors:** Ronan Lemwel Valdecantos, Michele Sorrentino, Michelangelo Mercogliano, Vincenzo Giordano, Ugo Trama, Maria Triassi, Raffaele Palladino

**Affiliations:** 1 Department of Public Health, University “Federico II” of Naples, Naples, 80138 Italy; 2 Global Health Workforce Network (GHWN) Youth Hub, World Health Organization, 1211 Geneva, Switzerland; 3 Department of Public Health, University “Federico II” of Naples, Via Pansini 5, Naples, 80131 Italy; 4 Department of Public Health, Experimental and Forensic Medicine, National Programme in One Health Approaches to Infectious Diseases and Life Science Research, University of Pavia, Pavia, 27100 Italy; 5 UOD 02 Prevenzione, Campania Region, Naples, 80143 Italy; 6 General Directorate of Health, Centro Direzionale C3, Campania Region, Naples, 80143 Italy; 7 Department of Primary Care and Public Health, School of Public Health, Imperial College, London, UK

**Keywords:** Human papillomavirus, Cancer, Vaccine, Vaccination programs, Sexually transmitted infection, Europe

## Abstract

The introduction of HPV vaccinations, that can prevent most prevalent HPV-related cancers of various body districts, is a public health milestone. Despite broad immunization programs, European Health Systems face structural and organizational difficulties that hinder care. This study examined structural and organizational elements that may affect HPV vaccine coverage. We searched numerous databases from January 1, 1995 to May 15, 2023, for literature on HPV immunization research methodologies. Structural and Organizational aspects that cause HPV vaccine concerns in women and men were examined in the outcome evaluations and the research examined vaccination willingness factors. Ottawa, JBI's critical appraisal tool, and Amstar quality assessment assessed bias. A total of 10 articles from 312 studies met the inclusion criteria. Studies were undertaken in Italy, Belgium, England, Switzerland, France, the UK, and Spain. There were also combined-diverse studies in 15 and 27 European countries. Several primary healthcare strategies have increased HPV vaccination rates. These include vaccine procurement and cost-effectiveness, school-based immunization programs, electronic health databases, health professional training, health education and communication, and monitoring and surveillance.

## Introduction

Globally, cervical cancer is one of the most prevalent cancers in women aged 15–44, but the human papillomavirus vaccine (HPVv) has raised hopes that cervical carcinoma can be eradicated soon, as multiple preventative cervical cancer vaccinations have been licensed in various countries [1, 2]. Structured Human papillomavirus vaccination programs have the capacity to prevent about 70% of cervical cancers and a considerable number of other HPV-related illnesses [3]. Since the years 2006 and 2007, HPVv has been introduced differently in each European country, depending on its underlying health care system and National Immunization Programme (NIP). The Cervarix® vaccine offers protection against high-risk oncogenic genotypes HPV 16 and 18, while the Gardasil® vaccine provides protection against low-risk oncogenic genotypes HPV 6 and 11, which are associated with the majority of genital warts. Gardasil® also includes protection against genotypes 16 and 18 [4]. The latest authorized vaccine, Gardasil9®, offers protection against nine strains of HPV: 6, 11, 16, 18, 31, 33, 45, 52, and 58. This novel vaccination provides protection against five supplementary carcinogenic types (HPV 31, 33, 45, 52, and 58) [5]. In 2008, the European Centres for Disease Prevention and Control (ECDC), comprehensive guidelines have been provided for the implementation of the HPV vaccination programme. These guidelines include the underlying reasons for vaccination, as well as a clear overview of the available evidence relating to the vaccine's efficacy, safety, and suggested target populations. [6, 7]. Surprisingly, although the overall incidence and mortality rates of cervical cancer have decreased significantly over recent years in the European Union and the European Economic Area (EU/EEA), there are still significant disparities between different racial, national, and ethnic groups. According to an article in Europe; HPV vaccination programs studied in their primary cohorts, 7 (13%) countries provide full or partial funding for gender-neutral vaccination (GNV) HPVv catch-up cohorts, thus offering equal access to protection against HPV-related cancer and diseases [6]. Fourteen years after the initial licensing of HPVv in 2006, fully funded HPV vaccination programs are provided for the primary cohort in 38 out of 53 (72%) countries included in the WHO/ER region [8]. As of 31 January 2020, 6 (11%) countries recommended HPVv without having implemented a program, and 7 (13%) countries still have no recommendation nor implementation of HPV immunization for childhood and adolescent populations [9, 10]. One of the members of the WHO Strategic Advisory Group of Experts on Immunization (SAGE) and its convening in April 2022 reiterated that: “we need political commitment complemented with equitable pathways for the accessibility of the HPV vaccine. Failure to do so is an injustice to the generation of girls and young women who may be at risk of cervical cancer.”

As of December 2021, all EU/EEA nations have included HPV vaccination into their national programs. In the next year, the Global plan suggested achieving the intermediate objectives for the three pillars of the elimination plan by 2030 as shown below — Vaccination: 90% of girls completely immunized with the HPV vaccine by age 15; Screening: 70% of women assessed with a high-efficacy test by age 35, and again by age 45; Treatment: 90% of women with pre-cancer were treated, and 90% of women with invasive cancer were managed [11]. By 2030, each nation must achieve the '90–70–90' objectives to be well positioned to eradicate cervical cancer [11]. The World Health Organization (WHO) advocated for the eradication of cervical cancer as a public health issue and established a goal of 90% HPV vaccine coverage by 2030 in the 2019 Global Strategy for the Elimination of Cervical Cancer as a Public Health Problem [12]. However, the HPVv mandate controversy has, in particular, revealed problems with the lack of consistent clear standards for the development of immunization policy. As the vaccine implementation and the quest for targeting coverage in Europe continues, systemic problems include the absence of registries to monitor individuals in need of vaccines, financial burdens associated with healthcare and vaccine expenses, limited healthcare accessibility in specific European regions, disparities in state vaccination laws and their interpretation, inadequate enforcement of existing requirements, inconsistency in guidelines, scarce health insurance coverage despite the presence of the Vaccination for Children (VFC) programme or the State Children's Health Insurance Programme (SCHIP), and insufficient dissemination of knowledge regarding vaccines [13]. Aside from the fact that organized screening works better than unorganized screening and that the European Council has made recommendations for cancer screening, health officials in eight former member states (Austria, Belgium, France, Germany, Greece, Luxembourg, Portugal, and Spain) have not yet started putting these recommendations into action on a national level for cervical cancer screening [14, 15]. Given the complexities of strategic vaccination and the diversified healthcare delivery system among European nations, this is a favorable time to evaluate measures and approach HPV vaccine and cervical cancer screening, and its acceptability and to consider the next steps to strengthen them. Hence, this article aims to (1) systematically review vaccination strategies used in published studies of the HPV vaccine; (2) examine studies on potential organizational and structural issues influencing HPV vaccination rates and describe their characteristics; and (3) suggest strategies to reach the HPV vaccination pragmatic target while marginalizing related cancer diseases as a primary health care tool.

## Methodology

### Criteria for inclusion and exclusion

Three fundamental criteria were necessary for inclusion: (1) Research articles published in English that presented original findings, including retrospective cohort studies, cross-sectional analyses, qualitative research, narrative literature reviews, and systematic reviews; (2) individuals aged 11 to 74 residing in the European Union (EU) and the European Economic Area (EEA) constituted our target demographic; (3) studies that provided data on our primary outcome and school-based metric: the percentage of the target population that had completed all recommended HPV vaccinations. The outcomes may pertain to single-dose vaccination, three-dose immunizations, and the cervical cancer screening now advised. We excluded papers without full paper availability or studies devoid of original data, including conference proceedings, editorials, or correspondence.

### Search strategy

We conducted an extensive search across multiple databases such as Cochrane CENTRAL, MedLine, EMBASE, PsycInfo, Psychology and Behavioural Sciences, and CINAHL–covering from 1 January 1995 to 15 May 2023; to identify articles that presented either quantitative or qualitative research on strategies aimed at increasing HPV immunization coverage. Only studies from the last 10 years that were significant were included. For the search strategy, keywords and Boolean operators were used, and each database was tuned to them, as explained in more detail; keywords for identifying Human Papilloma Virus using MeSH and key terms including “Papilloma”, “Human papillomavirus vaccine”, “HPV” and “HPV vaccine”. MeSH and key terms including “primary care”, “primary healthcare”; “school-based”, “organizational domain”, “organizational structure”, “immune”, “developed countries”, “developing countries”, and “Europe”, “European Union”, “EU” for the word Europe and to evaluate their effectiveness in the European region The European Economic Area (EEA). The outcome evaluations included organizational and structural aspects that lead to HPV vaccine apprehension. We ignored grey literature such as conference papers, dissertations, and government directives, focusing only on verified peer-reviewed literature.

The following strategy used for the search-related keywords and Boolean operators: (“Papilloma” or “Human papillomavirus vaccine” or “HPV vaccine”) OR (“primary care”, “primary healthcare”) OR (“school “ or “school-based”) OR ("developed countries" and "developing countries") OR (organi?ational structure in healthcare) OR ("vaccination coverage") OR ("european union" or eu or Europe”).

**Fig. 1:**
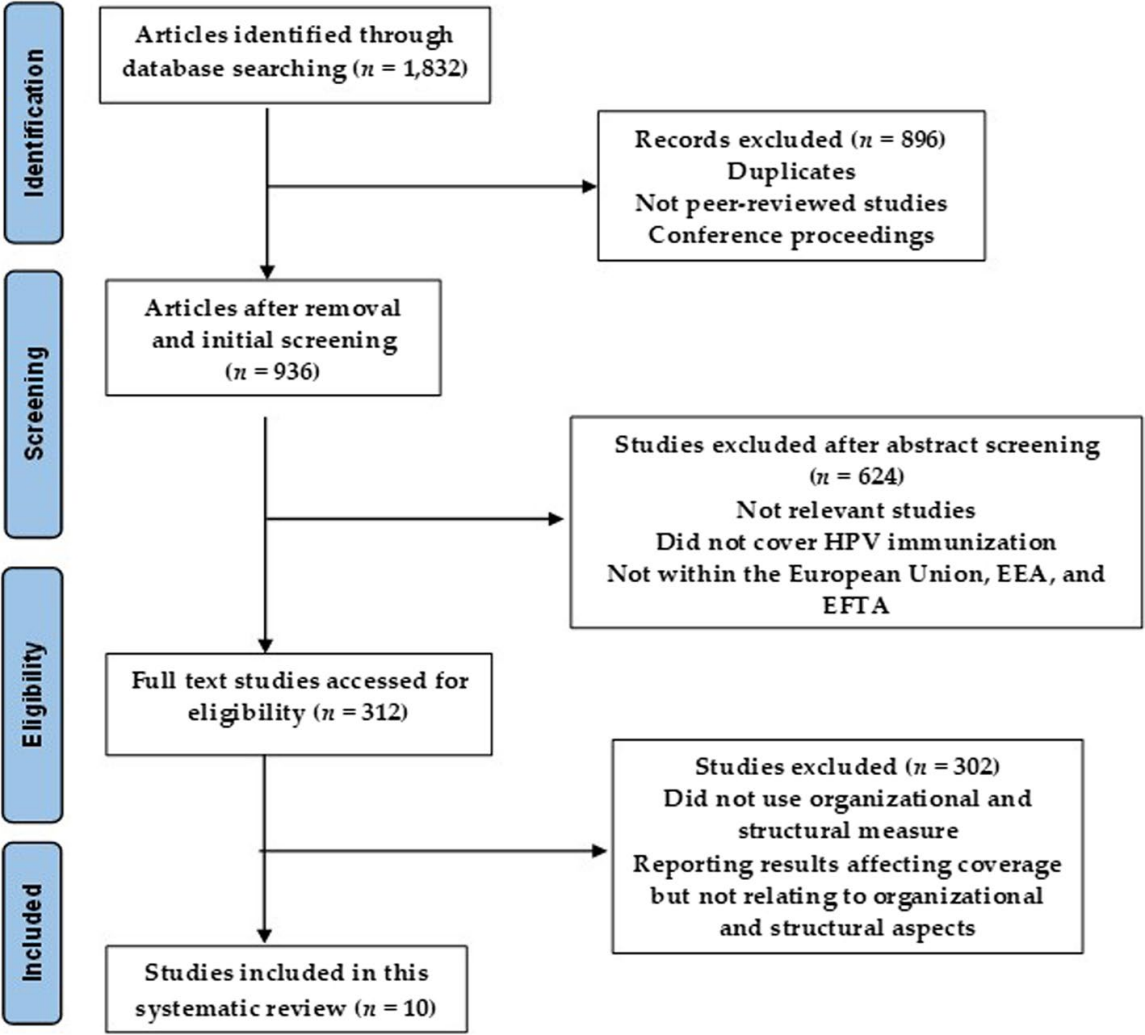
PRISMA flow diagram illustrates the search strategy and selection process

The selection procedure and the grounds for exclusion are depicted in the PRISMA diagram (Fig. 1). A researcher first reviewed the titles and abstracts of all publications identified by a search approach. Based on their titles and abstracts, all studies that did not address organizational and structural factors influencing HPV immunization coverage were excluded. Eligible full-text articles were obtained. Upon review, the validity of full-text articles regarding the link between structural and organizational aspects and vaccination coverage was established.

This systematic review was performed in accordance with the Preferred Reporting Items for Systematic Reviews and Meta-Analyses (PRISMA) guidelines. [16]. Electronic searches turned up a total of 1,832 papers; 896 and 634 of those studies were removed after their titles and abstracts were assessed, respectively. For a full-text review, the remaining 312 publications were retrieved. Ten manuscripts were found to be eligible for inclusion overall (see Fig. 1). Geographical location, age group, outcomes of interest, or unintended study design were the most frequently cited reasons for omission.

### Data extraction and quality assessment

One reviewer performed a comprehensive analysis of all citation titles and abstracts to ascertain their eligibility, using Rayyan Artificial Intelligence to pinpoint those that met the inclusion requirements. All instances of duplication were eliminated, and in cases where the abstract did not offer sufficient details to establish eligibility, the complete article was obtained for additional evaluation. Concerns about the articles were addressed through a discussion that involved the input of another reviewer. Moreover, an individual reviewer autonomously retrieved data from the studies that were included, focusing on the study's setting, characteristics of the participants, healthcare setting, interventions, and measurable outcomes. The methodological quality of the included studies was assessed using Ottawa, JBI's critical assessment tool, and Amstar, depending on the suitable study design.

## Results

### Characteristics of included articles

Table 1 presents a summary of the characteristics of the 10 selected studies. The 10 articles chosen were published from 2013 to 2023 [17–26]. Among the 10 studies reviewed, four were published between 2013 and 2016 [17, 19, 20, 25], another four from 2017 to 2020 [18, 21, 23, 24], and the remaining two were published from 2021 to 2023 [22, 26]. The research encompassed four cross-sectional studies [17, 22, 24, 26], two cohort studies [19, 21], two systematic reviews [18, 25], one qualitative study [17], and one narrative study [20]. Studies were carried out in Italy (2) 24,26], Belgium (1) [19], and England (1) [20], with the remainder conducted as combined-diverse studies in select 15 European countries (1) [18], Switzerland, France, the United Kingdom and Spain (1) [21], France (1) [22]and select 27 European nations [25]. The research was conducted in various settings including outpatient clinics, schools, educational facilities, family practices, primary healthcare clinics, community health centers, managed care organizations, health maintenance and related organizations, and community clinics across the European region and European Free Trade Association (EFTA).

**Table 1:** Study characteristics of included articles influencing HPV vaccine coverage

Study Characteristics of Vaccine Procurement and Cost-effectiveness							
Study		Quality	Type of Study	Setting /(Country)	Population/ (subject)	Intervention	Result / Outcomes
Qendri, 2018		10/10	Systematic Review study	European countries (Austria, Belgium, Croatia, Denmark, Estonia, France, Hungary, Iceland, Italy, Latvia, FYR Macedonia, The Netherlands, Norway, Poland, Portugal, Spain, Slovenia, Sweden, and the United Kingdom	Pricing of HPV vaccines	In several countries, the confidential nature of procurement agreements impedes transparency in vaccine pricing but providing access to procurement-based information; Tenders Electronic Daily (TED), the online version of the “Supplement to the Official Journal of the European Union” dedicated to the public procurement in Europe that aimed to: a. collect information on tender procedures for the HPV vaccines in European NIP’s (National Immunization Program) b. identify variables that are associated with HPV vaccine pricing in different tender-based settings	The average price per dose for the first-generation HPV vaccines decreased from €101.8 (95% CI 91.3–114) in 2007 to €28.4 (22.6–33.5) in 2017, whereas the average dose price of the 9-valent vaccine in 2016–2017 was €49.1 (38.0–66.8). Unit prices were, respectively, €7.5 (4.4–10.6) and €34.4 (27.4–41.4) higher for the 4-valent and 9-valent vaccines than for the 2-valent vaccine. Contract volume and duration, level of procurement (region or country), per capita GDP and number of offers received had a significant effect on vaccine price
Study Characteristics of Vaccine Procurement and Cost-effectiveness							
Study		Quality	Type of Study	Setting /(Country)	Population	Intervention	Result / Outcomes
Jit, 2015 [17]		9/10	Qualitative study	United Kingdom	Males and females aged 12–74 years old (cost-effectiveness analysis of two and three dose HPV vaccine	Examine the cost-effectiveness of vaccination, particularly when the duration of protection of two dose vaccination is assumed to be only 10 years, and hence the differencein reduction of cervical cancer between the two schedules is greatest (assuming no booster dose is administered at the point of warning)	Giving at least two doses of vaccine seems to be highly cost-effective across the entire range of scenarios considered at the quadrivalent vaccine list price of £86.50 (€109.23; $136.00) per dose. If two doses give only 10 years’ protection but adding a third dose extends this to lifetime protection, then the third dose also seems to be cost-effective at £86.50 per dose (median incremental cost-effectiveness ratio £17 000, interquartile range £11 700-£25 800). If two doses protect for more than 20 years, then the third dose will have to be priced substantially lower (median threshold price £31, interquartile range £28-£35) to be cost-effective. Results are similar for a bivalent vaccine priced at £80.50 per dose and when the same scenarios are explored by parameterizing a Canadian model (HPV-ADVISE) with economic data from the United Kingdom
Study Characteristics of School-based Vaccination program							
Study		Quality	Type of Study	Setting /(Country)	Population	Intervention	Result / Outcomes
Levefere, 2015		8/10	Retrospective cohort study	Belgium	Girls aged 12–18 years old	Determine whether after the encouragement program, the percentage of HPV vaccination initiation rates could then be compared to those realized under the free, school-based system and to know whether the reaction of the girls depended on their age	The personal information campaign significantly increased vaccination initiation, with older girls reacting faster. One year after the campaign the percentages of vaccination initiation for the oldest girls were 64.6 and 42.8% in the intervention and control group, respectively (*z* = 3.35, *p* = 0.0008); for the youngest girls the percentages were 78.4 and 68.1% (*z* = 1.71, *p* = 0.09). The combined personal information and financial incentive campaign increased vaccination initiation among certain age groups. One year after the campaign the difference in percentage points for HPV vaccination initiation between intervention and control groups varied between 18.5% (*z* = 3.65, *p* = 0.0002) and 5.1% (*z* = 1.12, *p* = 0.26)
Study Characteristics of School-based Vaccination program							
Study		Quality	Type of Study	Setting /(Country)	Population	Intervention	Result / Outcomes
Russell, 2013 [20]		5/10	Narrative Literature Review	England	Cost-effectiveness of Vaccine and the involvement of school nurses in the HPV vaccination program	Explore the health benefits of the HPV vaccine, the impact of attitudes, cost-effectiveness and the involvement of school nurses in programme delivery	The HPV vaccination, in conjunction with the screening programme, is an effective way of preventing cancer of the cervix. Although clinically beneficial to vaccinate young men, it is less effective for preventing cervical cancer than increasing vaccination rates among young women. Both bivalent and quadrivalent vaccines are equally effective and both show some cross protection for other HPV subtypes. The quadrivalent vaccine can prevent morbidity for genital wart type 6 and 11. A school-based programme has been shown to be effective with an uptake rate of 76% for 2009–2010, but this has implications for the role of the school nurse in delivering other services. With good communication, provision of accurate information and a flexible approach from healthcare professionals and parents, the uptake of the vaccination may improve. Due to high prevalence of HPV in young women, earlier screening is likely to result in high HPV detection rates and over-treatment of low-grade cervical disease
Study Characteristics of Education and Communication Strategies							
Study		Quality	Type of Study	Setting /(Country)	Population	Intervention	Result / Outcomes
Wymann, 2017		8/10	Cross-sectional study	Switzerland	Girls aged 18–49 years old	Measure uptake of and factors associated with human papillomavirus (HPV) vaccine such as: a. Determine HPV vaccination initiation and full coverage and factors associated with initiation of vaccination; reasons for not being vaccinated (to see if women know about the need for cervical cancer screening after HPV vaccination) b. Determine whether HPV vaccination affects adherence to cervical screening recommendations	Vaccination initiation was 69.3% and full coverage (three doses) 54.1% for 18–20-year olds, respectively, 42.4% and 33.9% for 21–24-year olds. Women with C 10 lifetime sexual partners were less likely to have received any HPV vaccination than women with B 2 partners (18–20 years OR 0.2, 21–24 years OR 0.5). Amongst 1000 unvaccinated women (18–24 years), reasons for not having initiated vaccination were lack of information (22.5%) and fear of vaccine side effects (18.1%). Vaccination status was not associated with adherence to cervical cancer screening recommendations (OR 1.3). 95.4% of all vaccinated participants knew about the continued need for screening
Study Characteristics of Education and Communication Strategies							
Study		Quality	Type of Study	Setting /(Country)	Population	Intervention	Result / Outcomes
Mari, 2022		8 /10	Cross-sectional study	Italy	Boys aged 6–18 years old	Examine either attitude toward HPV vaccination or the perceived need for more information on HPV, as well as their determinants while shedding light on the gap between expected and actual coverage and expect to inform future strategies in support of vaccination adherence	A positive attitude towards HPV vaccination was found in 74% of interviewed parents. Knowledge of HPV, having a generally positive attitude toward vaccination, and mothers filling in the survey were positively associated with a positive attitude to the HPV vaccine. Parents’ perceived need for more information about HPV vaccination was positively associated with the child’s age, general positive attitude toward vaccination, Christian religion, and positive attitude toward HPV vaccination; knowing that HPV vaccination is free of charge significantly reduced the risk of asking for more information on HPV vaccination
Study Characteristics of Surveillance and Monitoring							
Study		Quality	Type of Study	Setting /(Country)	Population/ (subject)	Intervention	Result / Outcomes
Carozzi, 2018		9/10	Cross-sectional study	Italy	Girls 11, 14, 17,24 years old (vaccine coverage and timing)	Assess vaccine and non-vaccine HPV prevalence 5–7 years post-vaccination program implementation in vaccinated and unvaccinated women	Overall, 2793 women (18–50 years) were included, 1314 of them having been in birth cohorts eligible for the HPV vaccination program (18- to 30-year-old women at enrolment). Among the latter, qHPV vaccine uptake was 59% (at least one dose), with 94% completing the schedule; standardized qHPV type prevalence was 0.6% in vaccinated versus 5.5% in unvaccinated women (P < 0.001); adjusted VE against vaccine type infections was 90% (95% CI: 73%−96%) for all fully vaccinated women and 100% (95% CI not calculable) in women vaccinated before sexual debut
Study Characteristics of Surveillance and Monitoring							
Study		Quality	Type of Study	Setting /(Country)	Population	Intervention	Result / Outcomes
Elfstrom, 2015		9/10	Systematic Review	27 countries from the European Union (EU) and European Free Trade Association (EFTA	HPV vaccination Programme	Identify how programs are currently organized, the costs associated with organizing and ensuring the quality of the program and how quality and effectiveness measurements are carried out	The majority of countries had some level of vaccination activity, with approximately half of the countries reporting an organized vaccination program. Centralized vaccine registries were in place in the majority of countries with an organized program, allowing for monitoring of key indicators at the national level. Costs of organization and monitoring were difficult to estimate and varied significantly, as some countries were able to use existing infrastructures while others had to create new systems, incurring greater costs
Study Characteristics of Electronic Health Database							
Study	Quality		Type of Study	Setting /(Country)	Population	Intervention	Result / Outcomes
Braeye, 2020	9/10		Retrospective Cohort study	Denmark, Italy, United Kingdom and Spain	The Accelerated Development of Vaccine Benefit Risk Collaboration Europe (ADVANCE)	Estimate the coverage with measles, HPV and influenza-vaccines using the Accelerated Development of Vaccine Benefit Risk Collaboration Europe (ADVANCE) (an e-HR-databases); a public private collaboration aiming to develop and test a system for rapid benefit-risk monitoring of vaccines in Europe	The age at the start of HPV-vaccination differed between databases as did the coverage attained at the age of 15 years. E.g. in the UK (THIN & RCGP-RSC) HPV-vaccination started one birth year later than in Denmark (SSI/AUH) and Spain (BIFAP). SSI/AUH attained a HPV-vaccine coverage of > 70% for birth year 1994 after which the coverage continued to increase. The other databases, except for RCGP-RSC and THIN, also reported an increasing coverage over the birth years included in this study. The attained HPVvaccine first dose coverage at the age of 15 years for females born between 1997 and 2000 estimated with the IPW-method ranged between 60% (RCGP-RSC/THIN) and 88.3% (SSI/AUH)
Study Characteristics of Health Professionals’ Training							
Study	Quality		Type of Study	Setting /(Country)	Population	Intervention	Result / Outcomes
Dib, 2022	9/10		Cross-sectional study	France	Girls aged 11–14 years old	Identifying factors associated with the uptake of HPV vaccine using Determinants of HPV Vaccine Hesitancy (FSQD-HPVH) based on French context This approach classifies the determinants of vaccine hesitancy into three main domains: (i) Contextual influences a. Communication and media environment b. Historical influences c. Religion/culture/gender/socioeconomic factors d. Politics/policies (ii) Individual and group influences a. Personal, family, and or community members’ experiences b. Beliefs and sttitudes about health and prevention c. Knowledge/awareness d. Health system and providers-trust and personal experiences e. Risk/benefit (perceived/heuristic f. Immunization as a social norm vs. not needed/harmful (iii) Vaccine/vaccination specific issues a. Introduction of a new formulation or a new recommendation for an existing vaccine b. Design of vaccination program/mode of delivery c. Vaccination schedule d. The strength of the recommendation and/or knowledge-based and/or attitude of healthcare professionals	Overall, 38.6% of the mothers indicated that their daughter received at least one dose of the HPV vaccine. The multivariate analysis revealed that agreeing with the statement that doctors/health care providers believe vaccinating girls against HPV was a good idea, and having asked questions to the attending doctor about HPV vaccines were associated with a higher HPV vaccine uptake (OR = 4.99, 95% CI [2.09–11.89]; and OR = 3.44, 95% CI [2.40–4.92]). Mother’s belief that her daughter was too young to be vaccinated against HPV (OR = 0.16, 95% CI [0. 09–0.29]) and lower daughter’s age (OR = 0.17, 95% CI [0.10–0.28] for girls aged 11 compared to those aged 14) were found strongly inversely associated with HPV vaccination, followed by agreeing with the statement that the HPV vaccine was unsafe (OR = 0.42, 95% CI [0.26–0.67]), identifying as true the statement that HPV was very rare (OR = 0.49, 95% CI [0.31– 0.77]), and the mother’s refusal of own vaccination (OR = 0.57, 95% CI [0.40–0.80])

### Vaccine procurement and cost-effectiveness

Two included studies focused on vaccine procurement and cost-effectiveness, one in EU countries and the other in the United Kingdom; both received high scores using Amstar2 for Systematic Review and the JBI Appraisal tool for Qualitative Study, respectively. The organization of HPV vaccination initiatives may also influence procurement outcomes, as well-organized programmes pose a lesser risk to both health authorities and vaccine manufacturers [17, 27, 28]. Contractual terms include factors anticipated to influence the ultimate unit price of purchase, including insurance costs, packaging options, and shipping fees [18]. Tenders Electronic Daily (TED) is the online version of the "Supplement to the Official Journal of the European Union," dedicated to public procurement in Europe. This website provides complimentary electronic access to bids published by EU institutions, agencies, and other authorities. It facilitates the identification and categorization of procurement notifications and awards by nation, region, and business sector [18]. Furthermore, since 2014, several national HPV vaccination programs have shifted from a 3-dose to a 2-dose regimen for preadolescent immunization, indicating a need for fewer doses. [17]. Economic modeling quantifies the relative value of various tactics in terms of their health and economic advantages given their potential consequences, allowing for an informed dose selection based on a risk–benefit analysis [17]. Two doses are anticipated to provide long-term protection, but whether it will be as long-lasting or wide as three doses is questionable [17]. It may be impossible to predict if two doses will protect for more than 30 years [17]. As a result, skipping the third dosage of the HPVv is risky because the results are unknown in comparison to continuing with three doses [17].

### School-based vaccination strategy

In the systematic review, we used proper quality evaluation techniques. Two studies about School-based vaccination strategy—one of them was high-quality study [19] and one was low-quality [20]. School-based, supplementary immunization is recognized as the most effective method for attaining elevated vaccine coverage, especially among underprivileged communities [19, 31]. The reason for this is that in a non-school based vaccination system, specific system-wide barriers may hinder people from initiating or successfully completing the immunization process [19]. Nevertheless, there is still uncertainty regarding which component of the integrated personal information-financial incentive campaign (the informative or the financial side) is responsible for the observed variations in vaccination behaviour [19]. Therefore, if governments make the decision to propose population-based HPV vaccination after thoroughly considering the health benefits and immunization risks supported by the existing evidence, a school-based delivery method proves to be significantly more efficient [20, 29]. Our analysis suggests that school-based HPV vaccination is more cost-effective than non-school based vaccination technique, considering the crucial significance of high vaccination coverage in achieving herd immunity [20, 30]. This conclusion holds true from both the payers' and societal viewpoints [19]. Estimating the additional costs and benefits of implementing these campaigns might be a valuable assessment in such cases [20]. The Joint Committee on Vaccination and Immunization (JCVI) advised that the optimal method for implementing the vaccination strategy was by means of utilizing schools. [20]. An increasing number of academy schools are being established, and as these schools have autonomy, they have the authority to regulate the presence of nurses during school hours [20]. The school-based programme had a 76% uptake rate for the 2009–2010 year, which has impacted on the responsibilities of the school nurse in providing additional services [20].

### Electronic health database

Using the Ottawa quality scale for cohort studies, one of the studies in the review about Electronic Health Database got a score of 9 out of 10 [21]. The ADVANCE project (an electronic health database; a collaboration between public and private entities, sought to accelerate the development and evaluation of a system for promptly monitoring the benefits and risks of vaccines [21, 32]. This system would utilize the existing healthcare databases in Europe [21]. The method used to estimate age-specific coverage is similar, which allows for its application in worldwide vaccine studies [21]. The validity of the proposed technique and the trustworthiness of eHR-databases as a source for coverage estimation were demonstrated through a comparison with published reference coverage estimates [21, 32]. Through this standard technique, the acquisition of the age-specific coverage estimates, as well as coverage estimates particular to calendar year or season granted to accessible electronic data [21].

### Health professionals’ training

One study in the evaluation on Health Professional Training earned a 9 out of 10 using the Ottawa quality scale for cohort studies [22]. Healthcare practitioners (HCPs), particularly family doctors, play a significant role in setting standards and are highly trusted by the public [22, 33]. Therefore, it is important to encourage mothers and guardians to consult them for assistance [22]. This could be accomplished by implementing a consultation programme targeted concerning mothers of 11-year-old girls [33]. During this programme, attending physicians would provide counseling to emphasize the significance of vaccinating their daughters against a highly prevalent virus [22]. The vaccine in question has been extensively tested and proven to be safe [22]. This emphasizes the need for training to enhance the health professionals' ability to provide recommendations for HPV vaccination, as well as the need for precise and consistent communication regarding the effectiveness of HPV vaccines [22, 33]. Furthermore, Effective health teaching can potentially provide valuable information for communication campaigns aimed at enhancing public awareness and understanding of HPV, its association with cancer, and methods of prevention [22, 34].

### Education and communication intervention

Two included studies on education and communication, one conducted in Switzerland and one in the United Kingdom [23, 27], obtained a high mark utilizing the Ottawa Appraisal tool for Cross-sectional study. Given the significant impact of knowledge about HPV on parents' inclination to vaccinate their sons, the perceived necessity for information regarding the HPV vaccine and its factors was acknowledged, which has received less attention in some studies [2, 27, 35]. The age of a child has a strong and adversely related to parents' perceived necessity for information [27]. These findings indicate that it may be beneficial to focus awareness-raising initiatives and informative discussions with paediatricians on parents of younger children [27, 35]. This approach can help promote earlier and more positive attitudes among parents towards HPV vaccination [27]. Understanding the factors behind individual decisions to not receive vaccinations might provide insights into strategies for enhancing vaccination rates and minimizing inequalities [23, 36]. Moreover, two prevalent reasons were mentioned for not receiving vaccinations were insufficient knowledge and apprehension regarding the potential negative effects of the vaccine. Addressing local disparities in HPV vaccination coverage, as well as addressing information gaps and uncertainties regarding HPV vaccine safety, will be crucial [23, 36].

### Monitoring and surveillance

Both monitoring and surveillance studies in the systematic review scored 9 out of 10 using Newcastle Ottawa Quality assessment for Cross-sectional and JBI (Joanna Briggs Institute) Quality assessment for Systematic review study. Organizing efforts to immunize against HPV have the capacity to substantially diminish the prevalence of HPV-associated diseases [25, 26]. If similar approaches are implemented for the coordination, surveillance, assessment, and enhancement of programmes, it could have a significant impact on the utilization of healthcare resources and the burden of disease in Europe [26, 37]. There are variances in the organization and standards of HPV vaccination programmes among different countries, and sometimes even within different parts of the same country [26]. The level of depth in the monitoring activities differs among programmes [26]. However, the participation of nations indicates a strong interest in evaluating and enhancing programme performance [37]. It is crucial to conduct a baseline analysis to understand the structure and implementation of programmes, as well as their monitoring and evaluation processes, in order to accurately measure the impact of immunization efforts [26]. Continuous surveillance of the occurrence of cervical pre-cancerous lesions and cancers, along with other HPV-related lesions including anal and oropharyngeal cancers, will be crucial in comprehending the overall cost-effectiveness and population-level advantages of HPV vaccines in preventing cancer [25, 37]. The concept of "unmasking" should be distinguished from type-replacement [25]. It is of equal significance to assess the test employed and guarantee its satisfactory performance [25]. Continuous monitoring is necessary to ensure the optimal performance of tests used for surveillance [25].

## Discusssion

The World Health Organisation (WHO) has released a plan for the period of 2022–2030 with the aim of eradicating cervical cancer in the European Region and currently, the worldwide supply of HPV vaccines currently satisfies the global demand [38]. However, the absence of adequate supply buffers may result in limitations on country-level availability in the next three years [39]. The EU also faces the consequences of this scarcity, particularly in its lower-income countries like Romania [40]. The primary cause of this shortage, as stated by the executive director of Vaccines Europe, is the rise in demand [41]. To overcome significant setbacks in achieving the 2030 elimination plan, the WHO's Strategic Advisory Group of Experts (SAGE) has proposed a temporary halt to HPV vaccination for boys. This is 26266intended to prioritize girls residing in countries with a high incidence of cervical cancer [41]. There has been a consistent period of stable demand for HPV vaccines lasting five years [40]. However, in 2018, the demand unexpectedly doubled. Quantifying the demand for HPV vaccines and tracking the number of vaccine shortages is challenging [38]. Furthermore, no government provides any data on the stock of HPV vaccines [41–43]. These issues affect today and tomorrow: establishing an EU strategy, creating targets and indicators when data is scarce and definitions vary; predicting shortages without national or international surveillance; responding to shortages while aiming for immunization targets in strategies/initiatives [42]. Also, the majority of European nations are disregarding this recommendation and are instead implementing a vaccination programme that is not biased towards any certain gender [41, 43]. Yet, under certain conditions, particularly in the setting of significant worldwide demand, there is no substitute for the acquisition coordinated by a coalition of nations and supranational institutions [44].

In all EU nations, HPV vaccination is funded by public health systems; yet, access to free-of-charge immunization differs based on the organization of each country's healthcare system [45]. Vaccines were acquired collectively by the nation and disseminated by local entities responsible for their administration, procured by physicians in private practices for subsequent administration, or individually purchased by patients at pharmacies and reimbursed by their health insurance providers [46]. A study across 31 European countries indicated that structured vaccination programs aimed at females in early adolescence and the provision of free vaccines were more prevalent in countries with high vaccination coverage rates (VCR) compared to those with very low VCR, suggesting these factors may be crucial for attaining elevated vaccine uptake [47]. Human Papillomavirus vaccination coverage rates (HPV-VCR) in regions with elevated vaccination coverage rates (VCR), HPV vaccinations were more frequently distributed systematically at the administration sites (9/10) compared to regions with significantly low VCR (2/4) [47].

The paucity of tender-based vaccination prices, particularly for novel vaccines, has lately been identified as a barrier to vaccine accessibility [46, 48]. Significantly, it is noted that competitive vaccination pricing can be attained via tender procedures, as vaccine costs are influenced by contract volume, duration, national context, per capita Gross Domestic Product (GDP), and the number of bids submitted. Annually, in Europe, the HPVv contract volume correlated with a reduction in the per-dose price of USD 13.21 (EUR 11.0) for every 100,000 doses [18, 49, 50]. Moreover, regional purchase of vaccines yielded elevated unit pricing, amounting to USD 10.68 (EUR 8.9) per dosage, in contrast to national procurement. Also, proposals from both manufacturers reduced the unit price of the vaccine by USD 5.40 (EUR 4.5) compared to procurement that got a single offer [18, 49, 50]. However, according to the regression research by Qendri et al., the initial procurement of the HPVv compared to subsequent purchases had a minimal impact on the vaccine price.

The price of each vaccine dose can vary significantly across different regions and may change over time [51]. In Italy, the vaccination programme varies significantly depending on the region [52]. This means that the age groups and number of people targeted for vaccination, as well as catch-up programmes and access procedures, can differ greatly [53]. The price cap for public healthcare providers has been established at €104.00, which aligns with the ex-factory price per dose that has been negotiated by the Italian agency for medicines. The nine-valent vaccination (Gardasil 9®) received approval from the Food and Drug Administration in December 2014 and from the European Medicines Agency in June 2015 [41]. This vaccine was developed to protect against nine HPV types (6, 11, 16, 18, 31, 33, 45, 52, and 58) [43, 44]. The nine-valent vaccination, given to both girls and boys, decreases the incidence of cervical cancer by 17%, anal cancer by 35% in men and 14% in females, and prevents over a million cases of genital warts over a century compared to the quadrivalent vaccine [44]. Shifting from the quadrivalent vaccine to the nine-valent vaccine for female-only immunization is cost-effective provided the price per dose does not exceed €201 [44]. In comparing standard bivalent and quadrivalent vaccinations to the newly available 9-valent vaccine, a two-dose human papillomavirus vaccine regimen seems to be the most efficacious choice, contingent upon the protection enduring for a minimum of 20 years. Close monitoring is essential, since the precise length of two-dose regimens may remain uncertain for many years [53]. On the contrary, when it comes to dealing with vaccine supply scarcity and attaining herd immunity, a study in India suggests that with the implementation of a single dose of HPVv and striving for widespread HPV vaccination, we can make significant progress towards eradicating cervical cancer more cost-effectively [53]. Research on immunogenicity, post-hoc analyses of efficacy trials, and post-licensure observational studies of females have shown that a single dose of the HPV vaccine is enough to make the immune system react in a way that protects against both the first and second infection with HPV [54].

Various locations have implemented different delivery strategies for HPVv introduction, including school-based programs targeting eligible girls based on age or grade. Given the limited familiarity with adolescent vaccines or school-based immunization programs in many settings, countries interested in utilizing school-based HPV vaccine programs would benefit from a summary of the existing country experiences implementing such programs [55]. A recent study in France concluded that School-based campaigns substantially increased vaccine coverage among all adolescents within a short period. Yet, underlying disparities in HPV vaccination were unchanged [55]. Moreover, counties that provided HPV vaccinations to catch-up groups in schools had the greatest vaccine uptake [56]. Counties providing vaccinations to girls in all schools and those administering vaccinations in select schools exhibited greater vaccine uptake than counties not offering vaccinations in any schools [57, 58].

To monitor vaccine effectiveness and safety, a source of data containing vaccination dates and types among the population, as well as clinical information on patients before and after vaccination; and importantly required for Pharmacoepidemiology and Pharmacovigilance (vaccine side effects and adverse events monitoring). To illustrate, Immunization information systems (IIS) are useful tools for monitoring vaccination coverage and estimating vaccine efficacy and safety [58]. Vaccines provided in primary care settings can be recorded in the Vaccination File for each patient using an internal record of vaccines relevant to each vaccine dose administered. Furthermore, the Spanish Database for Pharmacoepidemiologic Research in Primary Care (BIFAP) has longitudinal primary care electronic health records from nine Spanish areas, which are up to date for research as of 2001 and could be a valuable tool for conducting epidemiological studies on HPV vaccination [57]. BIFAP is currently working on a project to develop a systematic approach to assessing the eligibility of healthcare databases for vaccine studies, during which the coverage of several vaccinations and the incidence of various health outcomes in BIFAP are consistent with Spanish and European data. However, previous studies have not examined the accuracy and utility of HPV vaccine information in BIFAP [59]. According to a recent survey, the majority of BIFAP physicians and nurses believe that electronic vaccination records are required [59].

Highlighting the relevance of training, Healthcare Professionals feel more confident in providing advice after receiving appropriate training. Vaccinology should be introduced consistently into medical and paramedical curricula for both mandatory and optional vaccines. However, in general, training continues to prioritize cure over prevention [60]. To address the issue, the apparent lack of vaccinology training in the curriculum can be addressed by summer courses, followed by the inclusion of vaccinology modules. Additionally, vaccinators can receive post-academic training through symposia. HCPs can be educated to implement the announcement health teaching technique to increase the number of people who get vaccinated against HPV. It is recommended that the method be-Announce, Connect, Clarify, and Counsel, with the awareness that parents who initially say no may later change their minds and say yes. Consequently, vaccination should be something that is brought up once more at each following session or follow-up visit. Because of their critical function of serving as client and patient advocates, HCPs should be provided time to study through continuing (medical) education, with assistance from their professional societies. Many types of training should be available, including face-to-face settings, webinars, e-learning courses, conferences, and peer-to-peer learning, so that HCPs may select the most relevant format for themselves and feel more comfortable discussing HPV vaccine [61].

Educational initiatives have the potential to more easily affect the intention of adolescents and young adults to acquire HPV vaccination [62]. Consideration should be given to incorporating HPV education into the secondary educational curriculum in countries that have not yet implemented an appropriate HPV vaccination programme. The shared beneficial characteristics that influenced both sexes were the initiation of sexual activity and the general degree of knowledge about HPV and HPVv. Incorporating a specifically designed section on sexually transmitted diseases (STDs) into the general biology curriculum of high school could serve as a valuable teaching resource to enhance awareness among adolescents of school age. In addition, it appears that awareness-raising initiatives conducted via social networking and social media have a greater influence on individuals compared to those that utilize brochures and printed materials. Furthermore, it is emphasized that public health initiatives focusing on HPV and its immunization should specifically target men to increase their engagement in preventive healthcare [63].

A regional Italian study found that public health measures must be improved to promote HPV vaccination communication and awareness [64]. Both personal and culturally influenced elements have an impact on individuals' encounters with and inclination toward knowledge [65]. The findings of Petrova et al. (2015) indicate that Young women like transparent and upfront information about the dangers linked to the HPV vaccination. Similarly, when developing significant cross-cultural HPV vaccine programming, methods of disseminating information must also be taken into consideration in diverse populations that are difficult to reach using tailored information about the HPV vaccine [66]. However, certain aspects of the vaccination and how it was communicated resulted in feelings of uncertainty. To make progress in boosting HPV vaccine coverage, it is crucial to create interventions that are tailored to the individual context and aim to enhance confidence in HPV vaccination. Additionally, community involvement methods should be developed to foster public trust [67].

Given that HPV-associated malignancies and condylomas can now be prevented through vaccination, it is necessary to implement monitoring systems for these diseases that are comparable to those used for other vaccine-preventable diseases [68]. Process measures encompass the evaluation of the extent and effectiveness of immunization, screening, and treatment programmes, as well as the continual assessment of their quality and scope [69]. Monitoring the impact of vaccines on the population level requires a well-developed surveillance system and consistent resources, which may not be possible in many settings [70]. To further illustrate, in scenarios with constrained resources, it may be advantageous to prioritize the classification of tumors in younger women. Cervical malignancies in women under 50 are frequently associated with HPV strains 16 and 18. Focusing on this cohort may yield earlier effect data, as these women are closer to the required immunization age [71]. Furthermore, the monitoring of the impact of the HPV vaccine necessitates coordination among multiple sectors. A multi-collaboration between experts from various fields such as vaccination, cancer screening, cancer surveillance, infectious disease, virology, sexually transmitted infection, child and adolescent medicine, reproductive health, and policy-making bodies is necessary to address the methodological, clinical, and feasibility concerns related to monitoring different biological outcomes [72]. Although monitoring HPV prevalence is not deemed necessary for the implementation of HPV vaccination programmes [69], it can offer an initial assessment of effectiveness by measuring the extent of reduction in HPV prevalence. This can also provide potential evidence of herd immunity and cross-protection [73] if feasible. At the end of the day, Public health surveillance plays a crucial role in monitoring the progress toward achieving HPV-associated disease eradication.

## Strengths and limitations

Among the strengths of our systematic review; the following could be included: the search was performed on the most relevant databases, and the selection of studies and data extraction was performed by taking appropriate measures to prevent potential errors for selection bias. The novelty of this research is in our ability to systematically chart existing papers on the subject of investigation, thereby providing a comprehensive review that has not been undertaken before. We integrated research from various clinical and environmental contexts across many nations, enabling us to generalize the results within this framework. The disparities in quality, settings, service delivery, and intervention implementation among the included studies rendered meta-analysis challenging. Moreover, research conducted in languages other than English was excluded, representing a significant limitation of the study. Finally, the possibility of experimenter bias or human mistake cannot be eliminated due to the fact that data search, extraction, and risk of bias assessment were all performed by a single individual.

## Policy implications and future recommendations

Policymakers can use our findings to determine which set of interventions is necessary, and practitioners can use them to develop practice guidelines in healthcare and the health professions. It is suggested that a future comprehensive and methodical review be conducted, with this systematic review serving as a reference.

## Conclusion

A concerted effort is necessary to optimize uptake of the HPVv among adolescents and the targeted age-groups in the European region and EFTA. Overall, this review supports the use of environmental interventions such as school-based vaccination program. The said environmental approach consistently reached the greatest number of participants and achieved the highest vaccination rates. It shows that awareness-raising campaigns using social networking and social media have a stronger impact on people than those using leaflets and printed materials. When population-based immunization measures are not possible, we encourage various strategies that address both the healthcare and the patient. Moreover, the remarkable international success of government-initiated HPV vaccination programs should be used to inform and guide EU policy. Finally, a known barrier to successful vaccination is the 3-dose requirement. Early evidence indicates that fewer than 3 doses may be protective. To sum it up, Various methods have been discovered in our systematic research that may enhance HPV immunization rates in the European region and EFTA which include Vaccine procurement and cost-effectiveness, School-based vaccination program, Electronic Health Database, Health Professionals’ training, Health education and communication intervention, and monitoring and surveillance.

## Data Availability

The datasets used and/or analysed during the current study are available from the corresponding author on reasonable request.
